# Elusive *Angiostrongylus vasorum* infections

**DOI:** 10.1186/s13071-015-1047-3

**Published:** 2015-08-27

**Authors:** Angela Di Cesare, Donato Traversa, Simone Manzocchi, Silvana Meloni, Eleonora Grillotti, Edoardo Auriemma, Fabrizio Pampurini, Cecilia Garofani, Fabrizio Ibba, Luigi Venco

**Affiliations:** Faculty of Veterinary Medicine, University of Teramo, 64100 Teramo, Italy; Novara Day Lab – IDEXX Laboratories Italia, S.P. 9, 28060 Granozzo con Monticello, Italy; Veterinary Hospital “Città di Pavia”, Viale Cremona 179, 27100 Pavia, Italy; Veterinary Clinic “LidoVet”, Via Poggio di Venaco 16, 00121 Ostia, Italy; Veterinary Pratice “Centro Italia”, Via Biancifiori 3, 02100 Rieti, Italy; Istituto Veterinario di Novara, S.P. 9, 28060 Granozzo con Monticello, Italy; Bayer Sanità Animale, Viale Certosa 130, 20156 Milan, Italy; Veterinary Pratice “Poggio dei Pini” Strada 40, 09012 Capoterra, Cagliari, Italy

**Keywords:** *Angiostrongylus vasorum*, *Dirofilaria immitis*, Neoplasia, Diagnosis, Moxidectin

## Abstract

**Background:**

The parasitic nematode *Angiostrongylus vasorum* causes severe clinical signs in dogs. The disease is often challenging because infected animals are often presented with clinical signs overlapping those of other diseases.

**Methods:**

The present article describes six angiostrongylosis cases (Cases 1-6) that represent key examples of how canine angiostrongylosis may be extremely confounding. The six animals presented clinical signs compatible with canine angiostrongylosis but they were subjected to clinical examinations for other diseases (e.g. dirofilariosis or immune-mediated disorders) before achieving a correct diagnosis.

**Results:**

In Case 1 clinical, radiographic and ultrasound examinations' results resembled a lung neoplasia. Case 2 was a dog with a mixed infection caused by *A. vasorum* and *Dirofilaria immitis*. Case 3 was a critically ill dog presented in emergency for an acute onset of dyspnoea caused by lungworm infection. The dog died a few hours after presentation despite support and etiologic therapy. Case 4 was a dog presented for chronic hemorrhages and ecchymoses caused by thrombocytopenia of unknown origin, thought to have an inherited, immune-mediated or infective cause. Case 5 was referred for neurological signs due to a suspected discospondylitis. Case 6 was erroneously diagnosed infected only with *D. immitis* although the dog was infected only with *A. vasorum*. A timely administration of an anthelmintic (mostly moxidectin) showed to be effective in treating the infection in those dogs (i.e. Cases 1,2, 4 and 5) that did not suffer with severe lung haemorrhages yet.

**Conclusions:**

Dogs 1-5 were referred in two regions of Italy that are considered non-endemic for *A. vasorum*. These findings indicate that veterinarians should include angiostrongylosis in the differential diagnosis of cardio-respiratory distress also in non-endemic regions and should perform appropriate diagnostics in the presence of compatible signs even if the clinical picture is atypical.

## Background

*Angiostrongylus vasorum* (Nematoda, Metastrongyloidea) is a molluscan-borne parasitic nematode affecting the heart and pulmonary arteries of dogs and wild canids [1–3]. This nematode is present in well-known endemic foci of Europe (i.e. France, UK and Denmark) but it has been recently found in dogs living in regions previously considered free of infection [3–6], e.g. central and southern regions of Italy [7, 8]. At present, there is a clear indication for a geographic expansion of *A. vasorum* in countries of Europe. In infected dogs clinical signs range from a subclinical infection to severe, acute or hyperacute respiratory and cardiac distresses, coagulopathies, gastrointestinal and neurological disorders, with possible fatal outcomes [2, 5, 7, 9, 10]. The diagnosis is difficult because clinical, laboratory, and diagnostic imaging findings are not specific and subclinical or atypical pictures may occur [9, 11, 12]. A suspicion can be confirmed with the detection of L1s *via* the Baermann’s test [7] or of circulating antigens with a rapid kit [13].

However, many veterinarians are not vigilant about dog angiostrongylosis and these methods are not applied in several canine practices. It is important that the guard against this parasite is kept high in both endemic and previously free regions. The present article describes six unexpected cases of dog angiostrongylosis in Italy and discusses the importance of appropriate diagnostic approaches even in territories that are not considered endemic.

## Methods

### Case 1

**Fig. 1:**
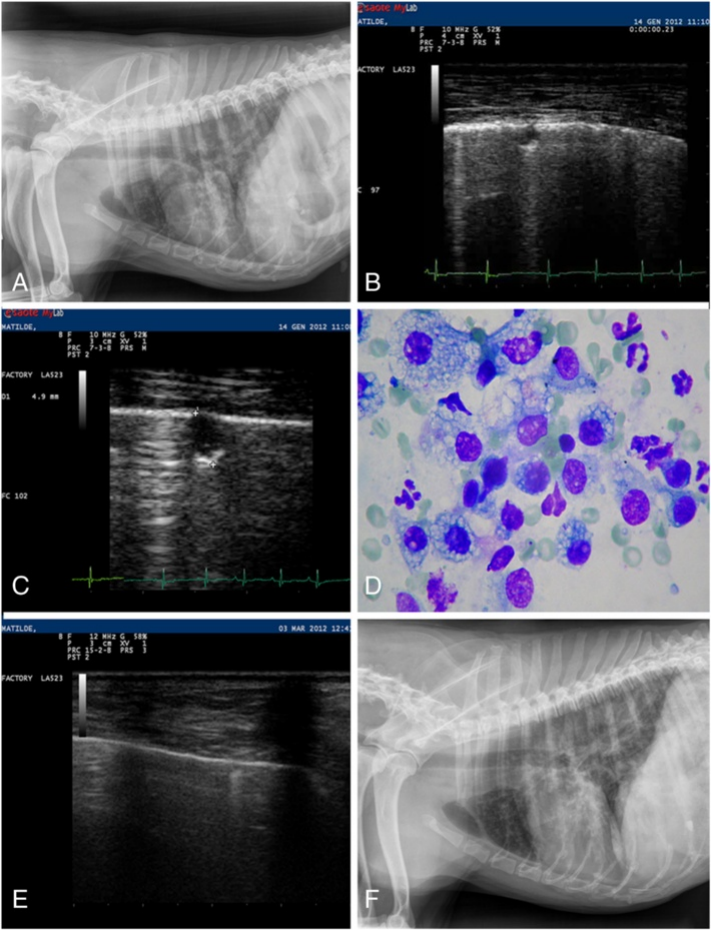
Case 1. **a** Thoracic radiograph, latero-lateral view. Normal cardiac silhouette, pulmonary interstitial pattern, presence of small nodules. **b** Pulmonary sonography. Scattered small sub pleural solid nodules. **c** Pulmonary sonography. Largest small sub pleural solid nodule, diameter ~5 mm. **d** Cytological examination. Mixed inflammatory cells, mainly foamy macrophage, with features of erythrophagocytosis. No bacteria and or other pathogens are present. **e** Post-treatment pulmonary sonography. No sub-pleural nodules can be visualized. **f** Post-treatment thoracic radiograph, latero-lateral view. Normal cardiac silhouette, mild pulmonary broncho- interstitial pattern, no more evidence of nodular lesions

A 2-year-old Dalmatian dog was referred to a private Veterinary Hospital in Pavia municipality (northern Italy, Lombardy region) with a history of respiratory distress and weight loss over the previous month and of unsuccessful therapy with ceftriaxone 50 mg/kg IM once a day for 2 weeks. The dog never moved outside of Pavia and lived outdoors. At admission the dog showed a mild dehydration and a severe dyspnoea. Haemocytometry and blood biochemistry were in normal ranges. Radiographic imaging of the thorax showed an interstitial pattern with no vascular lesions (Fig. 1a). Nodules of 2-7 mm of diameter in both lungs were visible at the pulmonary ultrasound examination (Fig. 1b,c). The cytological analysis of the nodules revealed the presence of macrophages, some of them showing erythrophagocytosis, and inflammatory cells (Fig. 1d). Overall, these findings were considered compatible with a pulmonary malignant disseminated neoplasia [14]. A routine copromicroscopic examination (i.e. floatation and Baermann’s test) showed the presence of several larvae of *A. vasorum*. The dog was treated with three doses of a spot-on solution containing imidacloprid 10%/moxidectin 2.5% (Advocate®, Bayer Animal Health) 15 days apart. In fact, after the first anthelmintic treatment the clinical status of the dog improved quickly, despite few *A. vasorum* L1s being shed in the faeces. Therefore, the dog received two further administrations of moxidectin. Forty-five days from the first treatment, the faeces were negative for *A. vasorum*, coughing and dyspnoea were absent, no nodular lesion could be revealed upon lung sonographic examination (Fig. 1e) and the thoracic radiographs were markedly improved (Fig. 1f).

### Case 2

**Fig. 2:**
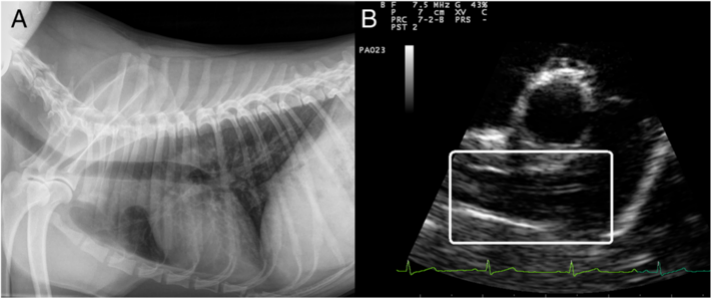
Case 2. **a** Thoracic radiograph. Latero-lateral view, right recumbency. No abnormalities of pulmonary vessels and cardiac silhouette. **b** Echocardiography. Right parasternal short axis view, slightly oblique for pulmonary trunk and right pulmonary artery optimal visualization. The pulmonary arteries do not appear dilated but filarid echoes are present (*white square*) within the right pulmonary artery

A 2-year-old mixed breed dog was referred at the aforementioned Hospital from a shelter, for a recent history of dyspnoea and coughing of mild entity of a few weeks duration. At the moment of the referral the clinical examination was normal, but the dog scored positive at the heartworm IDEXX Snap Test® and negative at the Knott’s test. Thoracic radiographs and echocardiography showed no relevant findings with the exception of filariid echoes in the right pulmonary artery (Fig. 2a and b). Therefore, a diagnosis of occult cardiopulmonary filariosis was made based on the positivity at the circulating antigen and the presence of heartworms in the pulmonary arteries.

Before starting with any treatment, the dog was subjected to a routine copromicroscopic examination that showed the presence of *A. vasorum* larvae in the faeces. Then, the animal was treated with Advocate® before initiating the treatment for cardiopulmonary filariosis. In the month after treatment with moxidectin the clinical signs gradually disappeared and, the Baermann examination was negative for *A. vasorum*. The echocardiography showed the persistence of filariid echoes in the right pulmonary artery without signs of pulmonary hypertension. After a few days, to avoid any risk of thromboembolisms, the dog underwent surgical removal of about ten heartworms and recovered completely.

### Case 3

**Fig. 3:**
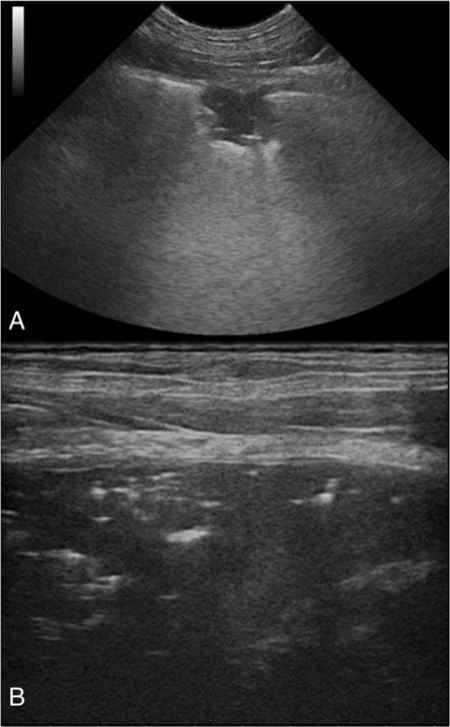
Case 3. Lung sonography. Small scattered well defined focal lesions into the subpleural pulmonary parenchyma (**a**) and lung consolidation (hepatization) of left caudal lung lobe (**b**) are shown

A 4-year-old, male Hannoverscher Schweisshund dog was referred to the same Hospital for severe respiratory distress and acute onset of dyspnoea. Hematological exam revealed a mild normocytic, normochromic, non-regenerative anemia in association with marked leucocytosis, marked absolute monocytosis, neutrophilia and left shift. Platelet and eosinophil concentrations were in the reference limits. Given the severe clinical picture and the hematologic abnormalities of the dog, an infective process, as a bacterial pneumonia, or a lung injury were first suspected. Radiographic evaluation of the thorax showed a marked generalized unstructured interstitial pattern. No sign of pulmonary hypertension could be detected, while lung sonography showed the presence of small scattered focal lesions in the pulmonary parenchyma and signs of hepatization of left caudal lung lobe (Fig. 3a and b). Diagnostic imaging lesions were suspect for a lungworm infection and specific tests for *A. vasorum* were performed. Evaluation of a direct fresh fecal smear revealed numerous nematode larvae morphologically compatible with *A. vasorum*. The infection was further confirmed by a positive antigenic test (IDEXX Angio DetectTM). After diagnosis the dog was immediately treated with a spot-on solution containing imidacloprid 10%/moxidectin 2.5% (Advocate®, Bayer Animal Health) in association with glucocorticoids and specific support therapy. The dog died six hours after therapy administration in a severe dyspneic status with profuse hemoptysis. Baermann’s test confirmed *A. vasorum* infection the day after.

### Case 4

**Fig. 4:**
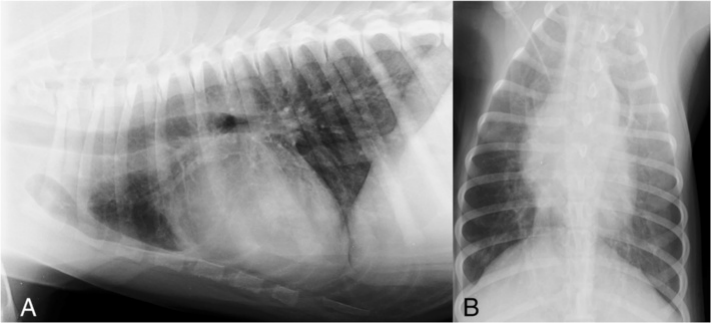
Case 4. RX image. Thoracic radiographs, latero-lateral view (**a**) and dorso-ventral view (**b**). Presence of a generalized unstructured interstitial to alveolar pattern; no evidence of cardiomegaly detected

A 6 month-old female Akita Inu was referred to a Veterinary Hospital in Novara municipality (northern Italy, Piedmont region) with a history of inappetence and chronic hemorrhages. Clinical examination revealed several foci of conjunctival hemorrhages, skin ecchymoses and petechiae, with no other clinical abnormality. Therefore, the first clinical suspect was an anticoaguant rodenticide poisoning. Nonetheless this suspicion was immediately discarded for the normal values of prothrombin time (PT) and activated partial thromboplastin time (aPTT). A complete blood count revealed a mild normocytic, hypochromic, non-regenerative anemia in association with moderate absolute monocytosis, moderate absolute eosinophilia and a severe thrombocytopenia, which was individuated as the cause of the primary hemostatic disorder showed by the dog. Radiographic examination of the thorax revealed a generalized unstructured interstitial to alveolar pattern compatible with lung hemorrhage or acute respiratory distress syndrome, which was considered the most probable differential due to the hemostatic disturbance of the dog (Fig. 4a and b). No evidence of cardiomegaly was radiographically detected. Therefore, an inherited, infective or immune-mediated cause of thrombocytopenia was suspected. Nonetheless, no predisposition for low platelet concentration is recognized in Akita Inu breed and specific tests for common infective or parasitic causes of thrombocytopenia (i.e. *Ehrlichia canis*, *Anaplasma platys*, *Anaplasma phagocytophilum*, *Babesia* spp.) were negative. The dog was then examined for *A. vasorum*, despite no records of this parasite existing in the Piedmont region of Italy. The dog tested positive at the IDEXX Angio Detect™ Test and this finding was confirmed by a Baermann test positive for *A. vasorum*. The dog was also positive at an antiplatelet antibody test. The dog was treated with fenbendazole 50 mg/kg sid for two weeks and tested negative for Baermann’s test two weeks after the end of the therapy. Suggested immunosuppressive approach was declined by the owner.

### Case 5

**Fig. 5:**
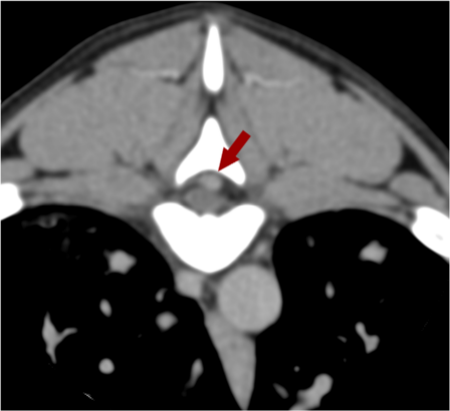
Case 5. CT transverse image of the thoracic spine. A endocanalar space-occupying hyperattenuating material (*arrow*) is seen and compressing the spinal cord

A 7-month-old female mix breed dog was referred to the same Veterinary Hospital of Case 4, with a history of anxiety, vocalizations, labored breathing in the previous day and an acute onset of paraplegia. Clinical examination revealed several neurologic deficits at the posterior limbs. Given the young age of the dog, the differential diagnoses included discospondylitis, myelitis or a compressive cause, with the former as the most likely diagnosis. Blood biochemistry and PT were in normal ranges and aPTT was slightly higher than the upper reference limits. Computed tomography (CT) of the vertebral column revealed an endocanalar hyperattenuating enhancing material with multifocal extra-dural distribution in the thoraco-lumbar tract compressing the spinal cord (Fig. 5). Moreover a diffuse interstitial lung pattern with enlarged and tortuous pulmonary arteries was seen. Despite first clinical suspects, diagnostic imaging findings make the clinician suspicious of *A.vasorum* infection as the cause of the lung lesion and pulmonary hypertension, therefore a rapid antigenic test (IDEXX Angio Detect™) for *A.vasorum* was performed giving a positive result. The endocanalar multifocal material seen in CT images was interpreted as hemorrhages or granulomatous lesions caused by larval dissemination of the lungworm. The infection was further confirmed positive by Baermann’s test. The dog was treated with fenbendazole 50 mg/kg sid for two weeks and tested negative for Baermann’s test two weeks after the end of the therapy. After the specific antiparasitic and support therapy, the clinical picture of the dog improved dramatically.

### Case 6

**Fig. 6:**
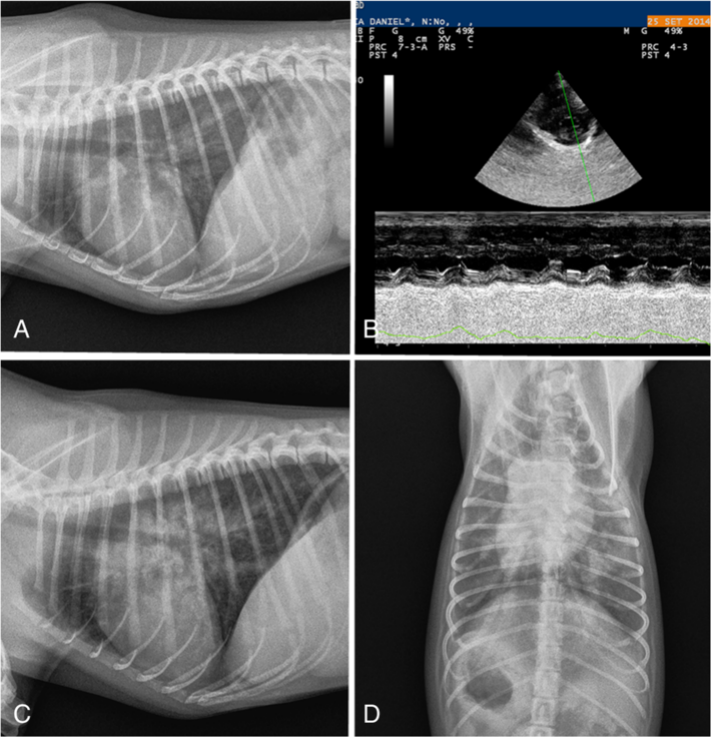
Case 6. **a** Thoracic radiograph, latero-lateral view. Presence of a bronchial pattern and no abnormalities of the cardiac silhouette. **b** Echocardiography, no abnormalities of cardiac chamber and valves. **c** and **d** Thoracic radiograph, latero-lateral (**c**) and dorso-ventral (**d**) views. Increase of radiopacity in peribronchial regions and alveolar pattern. No evidence of modification of the cardiac silhouette

**Fig. 7:**
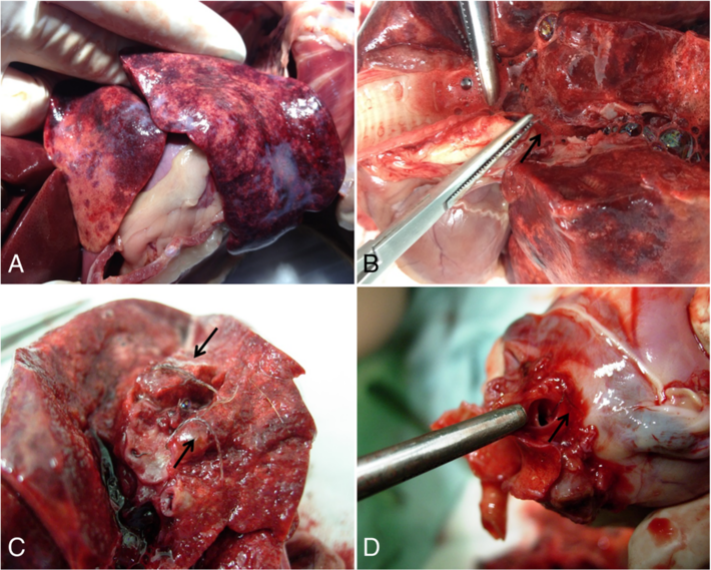
Case 6, Necropsy. **a** Large hemorrhagic areas at the lungs. **b** Presence of an abundant foam mixed with blood inside the trachea and main bronchi, and *Angiostrongylus vasorum* adult nematode picked up from a vessel (*arrow*). **c** Pulmonary hemorrhages, dark red cut lung surface and presence of *A. vasorum* adults (*arrows*). **d** Live *A. vasorum* in the lung vessels (*arrow*)

An 8-month old Pinscher dog was referred to a veterinary practice in Rieti municipality (central Italy, Lazio region) for a recent history of coughing. At the clinical examination, temperature, heart and lung sounds were in the normal ranges. Palpation of the trachea provoked wheezing and a persistent dry cough. After a diagnosis of tracheitis, the dog was treated with prednisone 1 mg/kg daily for 5 days and respiratory signs disappeared. After one month, the dog was again referred for relapse and worsening of respiratory signs and episodes of weakness of hind limbs. Auscultation of the lungs revealed crackles while the thoracic radiographs showed a bronchial pattern and an increase of the radiopacity of pulmonary vessels (Fig. 6a). No evidence of arrhythmia, cardiac heart failure or other abnormalities were present at the echocardiographic examination (Fig. 6b). The dog received a second daily course of anti-inflammatory and antibiotic drugs (i.e. prednisone 1 mg/kg and enrofloxacin 5mg/kg) but after three days it presented tachypnea, abdominal breathing and a heavy dyspneic crisis. Further radiographic examination demonstrated a serious increase of radiopacity in peribronchial regions, an alveolar pattern compatible with severe pulmonary edema or hemorrhage, and bulging of the main pulmonary artery in ventro-dorsal view (Fig. 6c and d). The dog was immediately examined for heartworms. The dog tested negative for microfilariae at the Knott’s technique while it scored weakly positive at the IDEXX 4Dx Plus®Test. Thus, a diagnosis of cardiopulmonary filariosis was made based on the positivity at this rapid test, the radiographic findings and the clinical signs, despite no echoes were recorded at the echocardiography and no microfilariae were found at the Knott’s examination. Before initiating any treatment the dog was subjected to a faecal examination which scored positive for *A. vasorum* L1s at both fresh faecal smears and Baermann’s test. Moreover the dog tested positive at the IDEXX Angio Detect™ Test. The dog was immediately treated with Advocate® but it died a few hours later of a severe pulmonary hemorrhage. At necropsy the chest cavity and the visceral pleura showed areas of thickening and fibrosis. The lungs presented diffuse hemorrhages at the caudal lobes and large hemorrhagic areas at the cranial lobes (Fig. 7a). Abundant foam mixed with blood was present in the trachea and main bronchi (Fig. 7b) and the cut surface of the bronchi was dark red, compatible with a pulmonary hemorrhage (Fig. 7c). Several live adults of *A. vasorum* were present in the main branches of the pulmonary artery and in the vessels of inferior caliber (Fig. 7d). No parasite or lesions were detected in the heart.

## Results and discussion

Although these dogs showed clinical signs compatible with angiostrongylosis, other differential diagnoses were chosen at first attempt. Therefore, it is indicated that dogs presenting a clinical picture compatible with angiostrongylosis should always be examined with appropriate diagnostic methods before excluding the infection even when other diseases are more plausible.

The clinical signs presented by the here examined dogs were due to the typical pathogenic mechanisms caused by *A. vasorum*, i.e. inflammation triggered by parasite eggs and larval stages in the lungs, and by damage caused by adult worms in the pulmonary vessels. In particular, infected dogs suffer from obstructed thrombotic endarteritis and fibrosis, and additionally, the parasite induces alterations of metabolic pathways (e.g. chronic Disseminated Intravascular Coagulation, DIC) [1–3, 5, 7–10]. In Case 1, clinical signs and Rx features were suggestive of a lung disease, including *A. vasorum*. However, veterinarians were misled to make another diagnosis by cytological examination and the absence of reports of this nematode in northern regions of Italy. In fact, the cytological findings of lesions were typical of lung haemangiosarcoma, that usually does not release neoplastic cells but only peri-tumoral tissue reaction [14].

The simultaneous presence of *A. vasorum* and *D. immitis* in Case 2 represented an intriguing case study as it provided a difficult diagnostic challenge. In fact *A. vasorum* was not suspected because history (i.e. the dog lived in a *D. immitis*-hyper-endemic region of northern Italy) and clinical findings were consistent with a diagnosis of cardiopulmonary filariosis. However, this dog had a subclinical occult dirofilariosis and the respiratory distress was due only to *A. vasorum*. Indeed, the similar localization of *A. vasorum* and *D. immitis* may cause overlapping clinical signs but *A. vasorum* is more prone than *D. immitis* to cause acute life-threatening diseases. Therefore, in this case an early diagnosis of angiostrongylosis was crucial to prevent the onset of a severe disease and death. Unfortunately, this was not the case of Case 6. In fact the detection of *A. vasorum* L1s coincided with a sudden worsening of the clinical condition of the dog. As moxidectin has a very high efficacy in treating dog angiostrongylosis [2, 3], the dog died from a severe lung hemorrhage that likely started hours before the administration of the anthelmintic. Interestingly, potential cross-reactions with *A. vasorum* in commercially available test kits for the diagnosis of filariosis were recently described for experimentally *A. vasorum*-infected dogs [15] but not for the Snap Test here used. This is the first evidence for a false positive reaction of the IDEXX 4Dx Plus® Test for *D. immitis* in a dog naturally infected with *A. vasorum*. Further studies are necessary to understand if this is an occasional finding due to the limit of specificity of the test (99.3% [16]); or more frequent cross-reactions may occur in clinical settings. On the other hand, it should be noted that the test was run using a sample of whole blood that could interfere with the reading phase of the test, thus causing difficulties in the interpretation of the result.

The critical presentation of Case 3 is not common for *A.vasorum* infection and the cause of the severe respiratory distress of the dog was not thought to be of parasitic origin until characteristic diagnostic imaging lesions put *A. vasorum* in the possible differential diagnoses. In this case a direct fecal smear proved useful in rapid detection of the lungworm considering the emergency situation of the dog. This copromicroscopical technique could be very helpful in emergencies, nevertheless a more sensitive and easy to perform test is now available for rapid detection of *A.vasorum* antigens on blood (IDEXX Angio DetectTM).

The hemostatic disorder of Case 4 was initially thought to be due to a poisoning but the finding of a severe thrombocytopenia led to a suspicion of inherited abnormal production of platelet or infective or haemoprotozoan diseases. Diagnostic imaging abnormalities observed in the thorax were compatible with hemorrhagic lesions and could fit easily the hemostatic disorder, thus masking the lungworms infection. Angiostrongylosis was not considered initially because this region of Italy is not considered endemic for *A. vasorum* and also because the clinicopathological picture of immune-mediated thrombocytopenia secondary to angiostrongylosis without evidence of intravascular coagulation is infrequent [17].

The atypical neurologic presentation of the dog n. 5 misled the clinicians to a discospondylitis or myelitis suspicion but a subsequent CT, performed to evaluate the spinal cord, revealed the coexistence of lung lesions compatible with *A. vasorum*.

## Conclusion

At present, dog angiostrongylosis is emerging in several areas due to biological and epizootiological drivers, e.g. global warming and changes in dynamics of intermediate hosts and fox populations [3, 4]. At the same time, *D. immitis*, which is hyper-endemic in Northern Italy, is spreading southward in Italy [7, 18] and also in European regions where also *A. vasorum* is expanding at the same time [3, 19]. Under a practical standpoint, the presence of clinical signs that could be present in both infections, should alert veterinarians to perform appropriate diagnostic techniques for both parasitoses. Angiostrongylosis should be further investigated also in dogs living in regions where the parasite is expected with null or low prevalence or even when more frequent diseases are suspected. The present report indicates that veterinarians are prone to exclude some diseases based on each local epizootiological situation. In fact, in Italy, dog angiostrongylosis is endemic in central and southern regions of Italy, while its occurrence in dogs living in the North is negligible and confined in certain areas [8, 20]. A prompt diagnosis of dog angiostrongylosis is crucial because, albeit the parasite may be life-threatening, the anthelmintic treatment is simple, straightforward and most often successful. In particular, a single application of moxidectin spot-on presents a high efficacy, i.e. similar to that observed in dogs treated with fenbendazole daily at 25 mg/kg for 20 days [21] and overlapping the efficacy achieved using milbemycin oxime (0.5 mg/kg) given orally once a week for 4 weeks [22]. Also, additional treatments (one or two, 15 days apart) with moxidectin are able to stop the larval shedding in dogs which are still infected after the first treatment, along with clinical and radiological improvements [8, 21, 23]. The cases here presented confirmed the efficacy of moxidectin spot-on and fenbendazole in treating *A. vasorum*. The death of dog n. 6 despite the administration of moxidectin was caused by the severity of the lung lesions and the pulmonary hemorrhages that in all likelihood started before the administration of the parasiticide. Under a practical point of view, it should be noted that easy-to-apply spot-on products are a suitable choice for the treatment of canine parasitoses when the alternative is represented by oral products requiring multiple dosing. In fact, the administration of oral formulations can be problematical in indocile, feral or moribund animals.

Considering that the current epizootiological changes will favor a spread of *A. vasorum* in previously free areas and an overlapping distribution of this nematode with that of *D. immitis*, a simultaneous use of highly specific diagnostic tools is crucial in both epizootiological surveys and clinical cases where these heartworms may occur and dogs present compatible clinical signs. Recently, an in-clinic rapid kit (IDEXX Angio Detect™ Test) has been marketed to achieve a fast and reliable diagnosis of the infection in veterinary practices [13]. The use of this kit, along with the Baermann’s test, would allow a fast and reliable diagnosis of dog angiostrongylosis and a timely anthelmintic treatment. The use of this rapid kit in routine screening of animals living in endemic areas is crucial especially for the occurrence of asymptomatic infected dogs [23].
